# A Novel Health Evaluation Strategy for Multifunctional Self-Validating Sensors

**DOI:** 10.3390/s130100587

**Published:** 2013-01-04

**Authors:** Zhengguang Shen, Qi Wang

**Affiliations:** School of Electrical Engineering and Automation, Harbin Institute of Technology, Harbin 150001, China; E-Mail: wangqi@hit.edu.cn

**Keywords:** health evaluation, data fusion, multifunctional self-validating sensor, health reliability degree, grey theory

## Abstract

The performance evaluation of sensors is very important in actual application. In this paper, a theory based on multi-variable information fusion is studied to evaluate the health level of multifunctional sensors. A novel conception of health reliability degree (*HRD*) is defined to indicate a quantitative health level, which is different from traditional so-called qualitative fault diagnosis. To evaluate the health condition from both local and global perspectives, the *HRD* of a single sensitive component at multiple time points and the overall multifunctional sensor at a single time point are defined, respectively. The *HRD* methodology is emphasized by using multi-variable data fusion technology coupled with a grey comprehensive evaluation method. In this method, to acquire the distinct importance of each sensitive unit and the sensitivity of different time points, the information entropy and analytic hierarchy process method are used, respectively. In order to verify the feasibility of the proposed strategy, a health evaluating experimental system for multifunctional self-validating sensors was designed. The five different health level situations have been discussed. Successful results show that the proposed method is feasible, the *HRD* could be used to quantitatively indicate the health level and it does have a fast response to the performance changes of multifunctional sensors.

## 1. Introduction

Multifunctional sensors have drawn more and more attention in modern production, because they can simultaneously detect several different parameters [1–3]. However, a multifunctional sensor will lead to a greater possibility of failure because it has more sensitive components [4]. Once faults occur, major industrial accidents could happen, so their health evaluation is extremely important.

**Figure 1.**
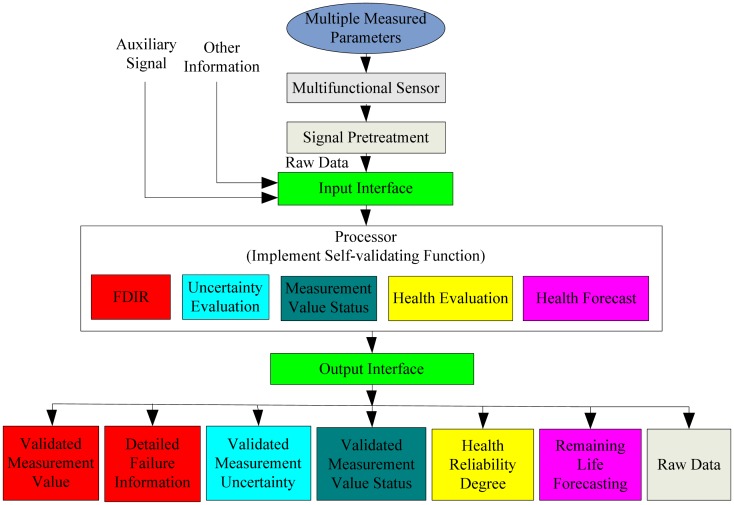
Functional architecture of a multifunctional self-validating sensor.

Aiming at the above problem, a multifunctional self-validating sensor model was proposed by authors [4,5] and its functional architecture is as shown in Figure 1. It not only includes traditional fault detection, isolation, and recovery (FDIR), but also provides the uncertainty of each measurement. Some previous work has been done [4–9], and this paper will center on the health evaluation to help users comprehend the current health level as well as the future performance degradation trend of multifunctional sensors.

The current approach to evaluate the health level of sensors is to use large numbers of experiments. These experimental setups are tested under different environmental parameters, such as temperature, humidity, pressure, power supply. The process is done by humans and it is very labor intensive. Another shortcoming is that humans may not be able to make out the relationships among the multiple variables of the multifunctional sensor. Further, some potential faults could happen too quickly for humans to detect them before they become catastrophic [10]. Most of existing automated methods only provide two health states (typically, healthy and faulty) [11–13], which is essentially a fault diagnosis. However, more detailed health information could not be obtained in this way, and a quantitative health evaluation may emerge as it can directly manifest the health level [10,14,15]. The vibration state is assessed in large capacity rotary machinery by using fusion information entropy [14], a health level of the liquid-propellant rocket engine ground-testing bed is given in [10], and a single sensitive component is preliminarily evaluated by using fuzzy set theory in [15]. The notion of quantitative health evaluation was mainly applied to complicated systems. Further, previous work centered on the health evaluation of single sensitive components and the method was also relatively complicated. Commonly, the correlations of multiple measured parameters are not fully used. From a quantitative point of view, the problem will become far more difficult and the quantitative health level analysis of multifunctional sensor not only involves the health level of each sensitive unit itself, but relates to their distinct weight distribution [16].

In this paper, we extend the traditional qualitative fault detection to quantitative health level evaluation by using multi-variable data fusion coupled with a grey evaluating algorithm [17,18]. It not only can be applied for fault detection, but also for health evaluation of multifunctional sensors. The interrelations of multi-variables can also be fully considered and this provides a health evaluation method from a “local” and “global” perspective.

This paper is organized as follows: Section 2 presents the proposed concept of health reliability degree; it is used as a quantitative index for the health condition evaluation of multifunctional sensors, while Section 3 discusses the novel methodology about how to evaluate the health level from asingle sensitive component and the overall multifunctional sensor. Section 4 designs a real experimental system of a multifunctional self-validating sensor and the actual samples of different health levels are used to verify the proposed methodology. Finally, Section 5 offers some concluding remarks and future directions.

## 2. Definition of Health Reliability Degree

**Figure 2.**
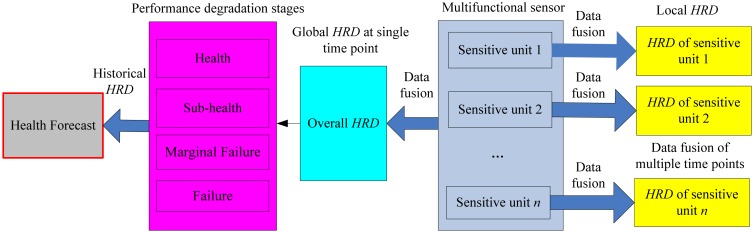
Health evaluation content of a multifunctional self-validating sensor.

The quantitative health levels to reflect sensor performance changes are implemented by using the proposed health reliable degree (*HRD*). Due to the presence of many more sensitive units, the health evaluation of multifunctional sensor not only includes each single sensitive unit but the overall sensor itself. The research content is shown in Figure 2.

Health levels of a single sensitive unit are different at different time points, so data fusion of multiple time points is needed to achieve the *HRD* of each sensitive unit as shown in Figure 2. The *HRD* of different time points can be treated as a tool for fault detection, and it should have fast response to faults. The overall health state of a multifunctional self-validating sensor is related to the importance of all sensitive units at certain time point, so its *HRD* can be obtained by using multiple sensitive unit data fusion as shown in Figure 2. Based on the *HRD* results, four degradation stages of sensor performance are defined and they are health, sub-health, marginal failure and failure. By using historical *HRD* information, health forecasting for multifunctional self-validating sensors can be done and this will play a more important role in industrial production. This thesis will emphasize the health evaluation aspect, and the health forecasting will be the topic of our next study.

The *HRD* is a comprehensive variable as a quantitative index, which indicates the degree of reliability of the multifunctional sensor and each sensitive unit. Health is an extent of degradation or deviation from an expected state, so the health evaluation is built on the expected health levels. Here, the expected sample can be acquired by calibration. Detailed descriptions about *HRD* are as follows:

### 2.1. Inner Meaning

The range of *HRD* is defined between 0 and 1. The state 0 indicates that the multifunction sensor or certain sensitive unit is in catastrophe failure mode, while state 1 is complete health. The different health levels are distributed between these extremes. The greater the value is, the higher the health level is. In this way, more detailed health information can be provided by using the proposed *HRD*, which benefits the understanding for users.

### 2.2. Extended Meaning

By using the *HRD* result, the four performance degradation stages of multifunctional sensor are defined as Health State (*HS*), Sub-Health State (*SH*), Marginal Failure State (*MF*), and Failure State (*FS*) respectively. Four classes of health levels are represented correspondingly. Here, the four stages can be also taken as the health features of a single sensitive unit. Further, the relationship between *HRD* and health degradation stages is defined in Table 1.

*HS*: The multifunctional sensor is very healthy. Each sensitive unit is also healthy and their measured data are nearly close to the true value.
*SH*: The multifunctional sensor is in sub-health and this is a state between *HS* and *MF*. The outputs of certain sensitive unit may fluctuate around their true values but within the normal ranges, so it is reliable to some extent. Commonly, it most situations multifunctional sensors are in *HS* or *SH*.
*MF*: The multifunctional sensor is nearly a failure. A few sensitive units are faulty, and their measured data have deviated from their true values, but none have deviated completely. Therefore, it is unreliable unless fault recovery is performed, which is also a topic of our future research.
*FS*: The multifunctional sensor is invalid. Most of sensitive units are faulty and the measurements have completely deviated from their true values, so it is totally unreliable.

### 2.3. Computation of HRD

The above four classes of health features are treated as four evaluating criteria of the grey evaluating model, and then their corresponding attached parameters are obtained. The computation of *HRD* can be further implemented by using the multi-variable data fusion of these parameters. In order to get the local and global *HRD*, the data fusion of a single sensitive unit among different time points and a single time point among multiple sensitive units are both needed, as shown in Figure 2.

**Figure 3.**
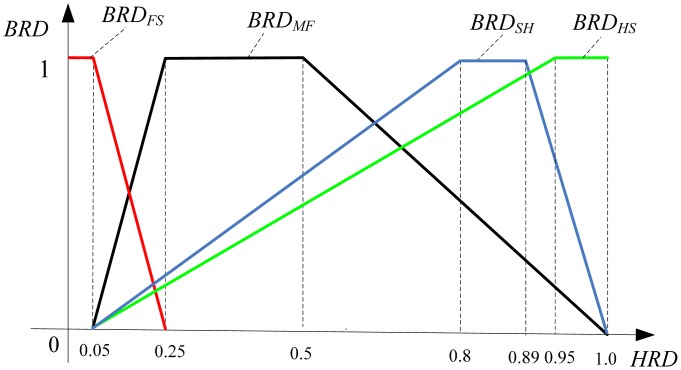
Relationship between *BRD* and *HRD*.

From the above analysis, the four attached parameters are the key to the computation of *HRD.* From Table 1, the attached degree of four evaluating criteria can be represented in a simplified way as shown in Figure 3. When the belonging relationship degree (*BRD*) to a certain criterion is the value 1, the current state is at its corresponding degradation stage. When the *BRD* is the value 0, the current state is completely not at its corresponding degradation stage. Other *BRD*s would decrease with the changes of *HRD*. To simplify the health evaluation problem, the decrease is assumed to be linear.

**Figure 4.**
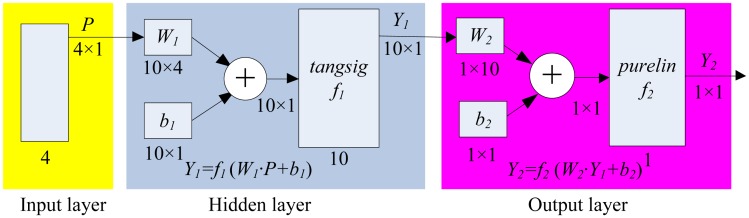
BPNN structure to fuse the *HRD* computing formula.

The computation of *HRD* involves mapping multiple variables, so acquiring such a clear formula to express the complex mapping is difficult. As one of the most promising technologies in computing, the back-propagation neural network (BPNN) is suitable to solve the health level problem. The input layer, hidden layer and output layer are included in BPNN and the formula of *HRD* is obtained by using the Matlab Neural Network ToolBox. The number of input layer nerve cells is *n* (*n* = 4) because we have four attached parameters *brd*_HS_, *brd*_SH_, *brd*_MF_, and *brd*_FS_, output layer cell *m* is 1 because of one desired *HRD*, and hidden layer has 10 cells according to experience formula $\sqrt{n+m}+a$ (*a* ∈ [1,10]), wherein *a* equals 7). The transfer function of the hidden layer is *tansig* (*f*_1_(*x*) = 2/(1 + *e*^−2^*^x^*)−1) and the output layer is *purelin* (*f*_2_(*x*) = *x*), and the Levenberg-Marquardt optimization based *trainlm* is selected as the training function because it is the fastest back-propagation algorithm in the toolbox. The BPNN structure is shown in Figure 4, wherein the *W*_1_, *b*_1_ represents the weight vector and threshold vector between input and hidden layer respectively, and corresponding *W*_2_, *b*_2_ between hidden and output layer.

In Figure 2, if the interval of *HRD* is defined as 0.01, *HRD*s between 0.05 and 1 can be divided into 95 blocks. If *HRD* is lower than 0.05, the health state is absolutely in *FS*. To decrease the number of training samples, the *HRD*s in *FS* are taken as zero. In summary, 96 training sample sets are selected, and the computing formula of *HRD* is then obtained by using the above BPNN. The formula can be written as in Equation (1) and the corresponding weight vectors and threshold vectors are also acquired:

(1) $$\begin{array}{ll} \text{HRD} & =f({\text{brd}}_{\text{HS}},{\text{brd}}_{\text{SH}},{\text{brd}}_{\text{MF}},{\text{brd}}_{\text{FS}})\\ & =f_{2}(W_{2}\cdot f_{1}(W_{1}\cdot P+b_{1})+b_{2}) \end{array}$$

where *P*=[*brd*_HS_, *brd*_SH_, *brd*_MF_, *brd*_FS_], *b*_2_=0.3834,

$$W_{1}=\left[\begin{array}{rrrr} -1.0005 & -1.7882 & 0.5971 & 1.1644\\ 1.0286 & -0.0875 & -0.2166 & -1.5229\\ 1.3250 & 0.1149 & -0.7423 & -1.2401\\ -1.1204 & -0.6091 & -1.2331 & -1.7552\\ 1.4452 & 1.2573 & 1.0365 & 0.4800\\ 0.9119 & -0.7922 & -1.4945 & 1.0284\\ -1.9908 & 0.9984 & 0.3054 & 0.7746\\ 0.8864 & -0.2861 & -2.3070 & -0.5203\\ -0.3461 & -1.6779 & 1.4637 & 0.6658\\ 1.7045 & -1.3901 & -0.8455 & 0.4996 \end{array}\right],b_{1}=\left[\begin{array}{r} 2.3165\\ -2.5580\\ -1.1886\\ 0.9639\\ -0.2892\\ 0.2948\\ -1.1430\\ 1.1854\\ -2.0245\\ 2.5613 \end{array}\right],W_{2}^{T}=\left[\begin{array}{r} 0.0739\\ 0.5939\\ 0.3065\\ -0.3890\\ 0.0851\\ 0.0050\\ -0.3522\\ -0.0336\\ -0.2433\\ -0.0169 \end{array}\right]$$

The *P* represents the degree of attached relationship to the criteria *HS*, *SH*, *MF*, and *FS* respectively. In Equation (1), the key issue of *HRD* computation is to solve the *P* and the detailed solution will be discussed in Section 3.

### 2.4. Significance Analysis

The mapping from actual measured data to the grey health level is implemented by using a grey algorithm while the de-greying process from grey evaluation to specific *HRD* is accomplished by using the proposed *HRD*. The definition of *HRD* has important significance in theory and practice.

The *HRD* is a basis of the health forecast. To predict the future health level of a multifunctional self-validating sensor, the current and past health states need to be understood. By using *HRDs* of different time points, the historical information could be collected and a time series constructed. The health prediction is done by using the time series analysis method, which benefits the understanding of the performance degradation in the real world.

The health level indicated by the proposed *HRD* can be directly understood by users and the corresponding precautionary measures can be taken to improve the sensor reliability. Taking the above four degradation stages as an example, if the sensor is in *HS* and *SH*, it works normally; the repair or data recovery is needed once it is in *MF*, and the sensor must be exchanged if it is in *FS*.

The computing method itself of *HRD* is open or extensible. By using the proposed idea, the evaluating criteria can be extended from four to more classes if necessary, and *HRD* results may be more concrete. The aim of this study is to present a new thought about health evaluation of multifunctional self-validating sensors.

## 3. *HRD* Methodology

**Figure 5.**
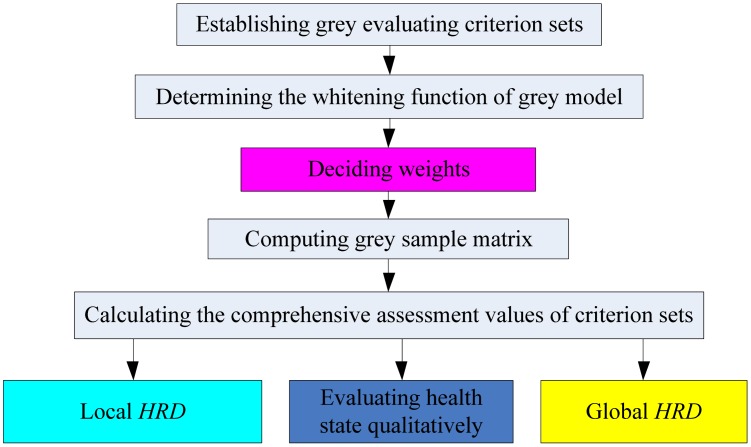
Flowchart of the *HRD* methodology.

The grey evaluation method coupled with the above *HRD* computing process is proposed to develop a new methodology for sensor health assessment. This novel strategy can provide a quantitative health level besides the traditional qualitative results. The corresponding flowchart is shown in Figure 5. The correlation among multiple parameters has been fully considered for the weight distribution of different sensitive units and time points, which is different from the traditional evaluation methods.

### 3.1. Establishing the Grey Evaluating Criterions

To distinguish the health hierarchy of multifunctional self-validating sensor accurately, four performance degradation stages (*HS*, *SH*, *MF*, and *FS*) are treated as the grey evaluating criteria sets.

### 3.2. Determining the Whitening Function of the Grey Model

**Figure 6.**
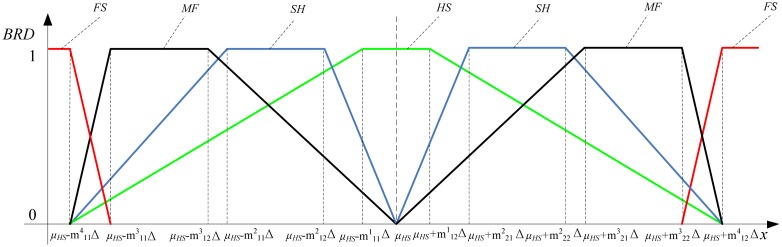
Relationship between *BRD* and measured value *x*.

The actual outputs of each sensitive unit have a mapping to the above four evaluating criteria sets. Some statistical researches on the multifunctional self-validating sensor have been done. It is a fact, that if a certain sensitive unit is not in *FS*, the measured outputs are closer to the true values, the grey *BRD* will become higher, and so will be the corresponding health level. The fact can be expressed in a more simplified way as shown in Figure 6. The *BRD* is 1 if the measurement *x* is within the allowed fluctuation range, while *BRD* decreases linearly if the *x* is outside the permitted range.

In application, the true value is difficult to obtain, its best estimation value *μ_HS_* can be acquired by using modern machine learning technology, for example, the data fusion result of multiple self-validating sensors or the mean value under health state can be taken as the best evaluated value. The allowed fluctuating interval is [*μ_HS_*−*m*^1^_11_Δ, *μ_HS_*+m^1^_12_Δ] if the *BRD* belongs to grey set *HS*. In a similar way, the grey intervals of other two grey sets *SH* and *MF* are defined as [*μ_HS_*−*m*^2^_11_Δ, *μ_HS_*-m^2^_12_Δ] and [*μ_HS_*+*m*^2^_21_Δ, *μ_HS_*+m^2^_22_Δ], [*μ_HS_*−*m*^3^_11_Δ, *μ_HS_*−m^3^_12_Δ] and [*μ_HS_*+*m*^3^_21_Δ, *μ_HS_*+m^3^_22_Δ] respectively. Here, the grey interval of grey sets *SH* and *MF* is symmetric.

In Figure 6, there is a premise that the sensitive unit is not faulty and the outputs are not completely unbelievable, so the above idea id only suitable for the evaluating criteria *HS*, *SH* and *MF*. The *FS* is under faults, so the continual use of the same idea is improper. The measurement *x* is completely unreliable if it is beyond the baseline *μ_HS_*−*m*^4^_11_Δ (or smaller than *μ_HS_*−*m*^4^_11_Δ) and *μ_HS_*+m^4^_12_Δ (or greater than *μ_HS_*+m^4^_12_Δ) as shown in Figure 6. If a certain sensitive unit is faulty, the *BRDs* under *FS* will undoubtedly become 1. Based on the above failure feature analysis, an upside-down trapezium is chosen as the whitening function under *FS*, as shown in red curve of Figure 6. In summary, the whitening function of four grey sets can be written in Equations (2–5) respectively:

(2) $$f_{\text{HS}}(x)=\{\begin{array}{ll} 0 & \begin{array}{ccc} x<\mu_{\text{HS}}-{m^{4}}_{11}\Delta & \text{or} & x>\mu_{\text{HS}}+{m^{4}}_{12}\Delta \end{array}\\ \frac{1}{{m^{4}}_{11}\Delta -{m^{1}}_{11}\Delta }[x-(\mu_{\text{HS}}-{m^{4}}_{11}\Delta )] & \mu_{\text{HS}}-{m^{4}}_{11}\Delta \leq x<\mu_{\text{HS}}-{m^{1}}_{11}\Delta \\ 1 & \mu_{\text{HS}}-{m^{1}}_{11}\Delta \leq x<\mu_{\text{HS}}+{{\text{m}}^{1}}_{12}\Delta \\ -\frac{1}{{{\text{m}}^{4}}_{12}\Delta -{{\text{m}}^{1}}_{12}\Delta }[x-(\mu_{\text{HS}}+{{\text{m}}^{4}}_{12}\Delta )] & \mu_{\text{HS}}+{{\text{m}}^{1}}_{12}\Delta \leq x<\mu_{\text{HS}}+{{\text{m}}^{4}}_{12}\Delta \end{array}$$

(3) $$f_{\text{SH}}\;(x)=\{\begin{array}{lll} 0 & & x<\mu_{\text{HS}}-{m^{4}}_{11}\Delta \;\text{or}\;x>\mu_{\text{HS}}+{m^{4}}_{12}\Delta \\ \frac{1}{{m^{4}}_{11}\Delta -{m^{2}}_{11}\Delta }[x-(\mu_{\text{HS}}-{m^{4}}_{11}\Delta )] & & \mu_{\text{HS}}-{m^{4}}_{11}\Delta \leq x<\mu_{\text{HS}}-{m^{2}}_{11}\Delta \\ 1 & & \mu_{\text{HS}}-{m^{2}}_{11}\Delta \leq x<\mu_{\text{HS}}-{{\text{m}}^{2}}_{12}\Delta \\ & \text{or} & \mu_{\text{HS}}+{m^{2}}_{21}\Delta \leq x<\mu_{\text{HS}}+{{\text{m}}^{2}}_{22}\Delta \\ -\frac{1}{{{\text{m}}^{2}}_{12}\Delta }(x-\mu_{\text{HS}}) & & \mu_{\text{HS}}-{{\text{m}}^{2}}_{12}\Delta \leq x<\mu_{\text{HS}}\\ \frac{1}{{{\text{m}}^{2}}_{21}\Delta }[x-(\mu_{\text{HS}}+{m^{2}}_{21}\Delta )] & & \mu_{\text{HS}}\leq x<\mu_{\text{HS}}+{m^{2}}_{21}\Delta \\ -\frac{1}{{m^{4}}_{12}\Delta -{{\text{m}}^{2}}_{22}\Delta }[x-(\mu_{\text{HS}}+{m^{4}}_{12}\Delta )] & & \mu_{\text{HS}}+{m^{2}}_{22}\Delta \leq x<\mu_{\text{HS}}+{m^{4}}_{12}\Delta \end{array}$$

(4) $$f_{\text{MF}}\;(x)=\{\begin{array}{ll} 0 & \begin{array}{ccc} x<\mu_{\text{HS}}-{m^{4}}_{11}\Delta & or & x>\mu_{\text{HS}}+{m^{4}}_{12}\Delta \end{array}\\ \frac{1}{{m^{4}}_{11}\Delta -{m^{3}}_{11}}[x-(\mu_{\text{HS}}-{m^{4}}_{11}\Delta )] & \mu_{\text{HS}}-{m^{4}}_{11}\Delta \leq x<\mu_{\text{HS}}-{m^{3}}_{11}\Delta \\ 1 & \mu_{\text{HS}}-{m^{3}}_{11}\Delta \leq x<\mu_{\text{HS}}-{{\text{m}}^{3}}_{12}\Delta \\ or & \mu_{\text{HS}}+{m^{3}}_{21}\Delta \leq x<\mu_{\text{HS}}+{{\text{m}}^{3}}_{22}\Delta \\ -\frac{1}{{{\text{m}}^{3}}_{12}\Delta }(x-\mu_{\text{HS}}) & \mu_{\text{HS}}-{{\text{m}}^{3}}_{12}\Delta \leq x<\mu_{\text{HS}}\\ \frac{1}{{{\text{m}}^{3}}_{21}\Delta }[x-(\mu_{\text{HS}}+{m^{3}}_{21}\Delta )] & \mu_{\text{HS}}\leq x<\mu_{\text{HS}}+{m^{3}}_{21}\Delta \\ -\frac{1}{{m^{4}}_{12}\Delta -{{\text{m}}^{3}}_{22}}[x-(\mu_{\text{HS}}+{m^{4}}_{12}\Delta )] & \mu_{\text{HS}}+{m^{3}}_{22}\Delta \leq x<\mu_{\text{HS}}+{m^{4}}_{12}\Delta \end{array}$$

(5) $$f_{\text{FS}}\;(x)=\{\begin{array}{ll} 1 & \begin{array}{ccc} x<\mu_{\text{HS}}-{m^{4}}_{11}\Delta & or & x>\mu_{\text{HS}}+{m^{4}}_{12}\Delta \end{array}\\ -\frac{1}{{m^{4}}_{11}\Delta -{m^{3}}_{11}\Delta }[x-(\mu_{\text{HS}}-{m^{3}}_{11}\Delta )] & \mu_{\text{HS}}-{m^{4}}_{11}\Delta \leq x<\mu_{\text{HS}}-{m^{3}}_{11}\Delta \\ 0 & \mu_{\text{HS}}-{m^{3}}_{11}\Delta \leq x<\mu_{\text{HS}}+{m^{3}}_{22}\Delta \\ \frac{1}{{m^{4}}_{12}\Delta -{m^{3}}_{22}\Delta }[x-(\mu_{\text{HS}}+{m^{3}}_{22}\Delta )] & \mu_{\text{HS}}+{m^{3}}_{22}\Delta \leq x<\mu_{\text{HS}}+{m^{4}}_{12}\Delta \end{array}$$

The matrix form of some parameters in Equations (2–5) can be represented as:

$$\begin{array}{cccc} M_{1}=\left[\begin{array}{cc} {m^{1}}_{11} & {m^{1}}_{12} \end{array}\right], & M_{2}=\left[\begin{array}{cc} {m^{2}}_{11} & {m^{2}}_{12}\\ {m^{2}}_{21} & {m^{2}}_{22} \end{array}\right], & M_{3}=\left[\begin{array}{cc} {m^{3}}_{11} & {m^{3}}_{12}\\ {m^{3}}_{21} & {m^{3}}_{22} \end{array}\right], & M_{4}=\left[\begin{array}{cc} {m^{4}}_{11} & {m^{4}}_{12} \end{array}\right] \end{array}$$

The degree of the deviation from the best estimation *μ_HS_* is assumed to be symmetrically distributed, which could avoid more parameter settings. The parameter Δ itself is only a base-level. Taking a temperature sensitive unit for example, if the best estimation value is 20 °C, and the base-level standard Δ is 0.01, the grey interval of *HS* would become [0.1, 0.1] when *M*_1_ is defined as [10, 10].

### 3.3. Computing Grey Sample Evaluating (GSE) Matrix

By using the above established whitening functions of different evaluating criteria, the *GSE* matrix can be obtained. The computation of *HRD* includes a single sensitive unit and the overall multifunctional self-validating sensor, and their meanings are provided here.

The *GSE* matrix of a single sensitive unit at multiple time points is denoted as *GSE_i_* = (*gse_ijk_*)*_m_*_×_*_n_* (*j* = 1, 2, …, *m; k* = 1, 2, …, *n*) as shown in Equation (6):

(6) $$GSE_{i}=\begin{array}{c} \begin{array}{llll} I_{1} & I_{2} & \cdots & I_{n} \end{array}\\ \begin{array}{c} T_{1}\\ T_{2}\\ \vdots \\ T_{m} \end{array}\left[\begin{array}{cccc} a_{i11} & a_{i12} & \ldots & a_{i1n}\\ a_{i21} & a_{i22} & \ldots & a_{i2n}\\ \vdots & \vdots & \vdots \\ a_{\text{im}1} & a_{\text{im}2} & \ldots & a_{\text{imn}} \end{array}\right] \end{array}$$

where *i* represents the certain sensitive unit, *T_j_* (*j* = 1, 2, …, *m*) is the different time point, and *I_k_* (*k* = 1, 2, …, *n*) is the evaluating criterion, it refers to the *HS*, *SH*, *MF*, *FS* in this paper.

The *GSE* matrix of multiple sensitive unit at single time point can be expressed as *GSE_j_* = (*gse_ijk_*)*_m_*_×_*_n_* (*i* = 1, 2, …, *m; k* = 1, 2, …, *n*) as shown in Equation (7):

(7) $$GSE_{j}=\begin{array}{c} \begin{array}{llll} I_{1} & I_{2} & \ldots & I_{n} \end{array}\\ \begin{array}{c} S_{1}\\ S_{2}\\ \vdots \\ S_{m} \end{array}\left[\begin{array}{cccc} a_{1j1} & a_{1j2} & \ldots & a_{1jn}\\ a_{2j1} & a_{2j2} & \ldots & a_{2jn}\\ \vdots & \vdots & \vdots \\ a_{mj1} & a_{mj2} & \ldots & a_{mjn} \end{array}\right] \end{array}$$

where *j* represents the time point, *S_i_* (*i* = 1, 2, …, *m*) indicates all the sensitive units of the multifunctional sensor, and *I_k_* (*k* = 1, 2, …, *n*) is still the evaluating criterion.

### 3.4. Deciding Weights

The analytic hierarchy process (*AHP*) [19–22] and objective information entropy [23] are the common methods used to decide weights. As for the *HRD* of a single sensitive unit at multiple time points, its outputs will change with the passage of time, such as the drift fault. The arbitrary pair-wise comparison among the time points is unavoidable, so the *AHP* is very suitable to amplify the importance of certain time point, for example, the moment when a fault occurs brings larger weight. As for *HRD* of the overall multifunctional self-validating sensor, the health evaluation assignment is implemented at a single time point, and the comparison among multiple time points is meaningless. It only needs the objective weight of each sensitive unit to indicate its information importance, which can be well expressed by using the information entropy method. Detailed application in sensor health evaluation is as follows.

#### 3.4.1. Computing the Weights of Different Time Points by Using AHP

Before the further computation of *HRD*, the guideline of computing weights is proposed as follows:

##### Guideline

The farther the output of a certain sensitive unit *i* deviates from its best estimation *μ_HS_* at certain time point *j*, the greater the measured value *x* brings importance to this sensitive unit.

If the measurement at certain time point is closer to *FS*, its sensitivity will be higher. In this way, the sensitivity of different time points is expressed by the weight *W_i_* = (*w*_i1_, *w*_i2_, …, *w*_ij_, …, *w*_i_*_m_*) and its calculation process is as follows:

Firstly, based on the proposed guideline, the scaling value *d_ij_* of sensitive unit *i* at time point *j* is defined as:

(8) $$d_{\text{ij}}=\left|x_{\text{ij}}-\mu_{\text{ij}}(\text{HS})\right|$$

where *x_ij_* is the actual output of sensitive unit *i* at the time point *j*, *μ_ij_*(*HS*) is its best estimation. Then the scaling values *d_i_* = (*d_i_*_1_, *d_i_*_2_,…, *d_ij_*,…, *d_im_*) of sensitive unit *i* could be derived and *m* is the number of time points.

In Equation (8), the *d_ij_* becomes greater if the difference between the actual value and *μ_ij_*(*HS*) is larger. In other words, if the measured value is closer to the *FS*, the corresponding time point is of greater importance, which conforms to the above guideline.

Secondly, construct the comparison matrix *CM_i_* in which each element is used to compare with others as shown in Equation (9). Its physical meaning is a relative importance comparison between two arbitrary time points:

(9) $$CM_{i}=\left[\begin{array}{cccc} \frac{d_{i1}}{d_{i1}} & \frac{d_{i1}}{d_{i2}} & \cdots & \frac{d_{i1}}{d_{im}}\\ \frac{d_{i2}}{d_{i1}} & \frac{d_{i2}}{d_{i2}} & \cdots & \frac{d_{i2}}{d_{im}}\\ \vdots & \vdots & \cdots & \vdots \\ \frac{d_{im}}{d_{i1}} & \frac{d_{im}}{d_{i2}} & \cdots & \frac{d_{im}}{d_{im}} \end{array}\right]$$

Thirdly, solve all the eigenvalues *λ* and eigenvectors *α*, and then pick out the largest eigenvalue *λ*_max_ as well as its corresponding eigenvector *α*_max_:

(10) $${\text{CM}}_{i}\;\alpha =\lambda \;\alpha$$

Fourthly, check its consistency. In order to test the validity of the weights distribution generated by using *AHP*, the consistency index (*CI*) and the overall consistency ratio (*CR*) are calculated by using Equation (11). If *CR* is lower than 0.10, the weights distribution will be acceptable:

(11) $$\begin{array}{c} \text{CR}=\frac{\text{CI}}{\text{RI}}\\ \text{CI}=\frac{\lambda_{\max}-n}{n-1} \end{array}$$

where *n* is the order of the *CM_i_*, and average random consistency variable (*RI*) is shown in Table 2 when *n* is lower than 15 over 1,000 experiments.

Lastly, the eigenvector *α*_max_ is taken as the weight vector *W_i_*, which represents the relative importance of different time points.

#### 3.4.2. Computing the Weights of Different Sensor Units by Using Information Entropy Method

The objective weight of each sensitive unit is obtained as follows:

Firstly, construct the grey evaluating matrix *GSE* = (*gse_ik_*)_m×n_ as shown in Equation (7).

Secondly, compute the *k*th assessment criterion's probabilistic proportion *P_ik_* of *i*th sensitive unit by using Equation (12):

(12) $$\begin{array}{cc} P_{\text{ik}}=\frac{{\text{gse}}_{\text{ik}}}{\sum_{k=1}^{n}{\text{gse}}_{\text{ik}}} & \left(i=1,2,\cdots ,m;k=1,2,\cdots ,n\right) \end{array}$$

Thirdly, compute the information entropy *E_i_* of the *i*th sensitive unit by using Equation (13):

(13) $$\begin{array}{ll} E_{i}=-\frac{1}{\ln n}\sum_{k=1}^{n}P_{\text{ik}}\ln P_{\text{ik}} & \left(i=1,2,\cdots ,m\right)\\ \text{subject to}P_{\text{ik}}\ln P_{\text{ik}}=0 & \text{if}P_{\text{ik}}=0 \end{array}$$

Fourthly, compute the deviation *G_i_* by using Equation (14). The greater the output *G_i_* is, the more important the sensitive unit is:

(14) $$\begin{array}{cc} G_{i}=1-E_{i} & \left(i=1,2,\cdots ,m\right) \end{array}$$

Lastly, compute the objective weight *w_ij_* of the *i*th sensitive unit at the time point *j* by using Equation (15) and then the weight vector *W_j_* = (*w_1j_*, *w_2j_*, …, *w_ij_*, …, *w_mj_*) is determined correspondingly:

(15) $$\begin{array}{cc} w_{\text{ik}}=\frac{G_{i}}{\sum_{i=1}^{m}G_{i}} & \left(i=1,2,\cdots ,m\right) \end{array}$$

### 3.5. Calculate the Comprehensive Grey Assessment Values Under Evaluating Criterion Sets

The weights *W_i_* of a single sensitive unit *i* at multiple time points and *W_j_* of the multifunctional self-validating sensor at single time point *j* have been obtained, then the comprehensive grey assessment values (*CGAV*) are computed using Equation (16). The *CGAV* represents the current health distribution under the above four grey health evaluating criteria sets, and they are exactly the feature attached parameters in Equation (1):

(16) CGAV=W×GSE

In Equation (16), the following introduction should be made:

1. When both *W* and *GSE* represent multiple sensitive units at certain time point *j*, the fused *CGAV* indicates the grey assessment values of the overall multifunctional self-validating sensor.
2. When both *W* and *GSE* represent a single sensitive unit *i* at multiple time points, the corresponding *CGAV* would be fused into the grey assessment values of the certain sensitive unit *i*.

### 3.6. Computing HRD

The proposed health evaluation strategy can provide the qualitative assessment result as usual. The health state belongs to the health evaluating criterion which has the maximum of *CGAV*s. From a local and global perspective, the *HRD* is then calculated to describe the detailed health information by using Equation (1).

## 4. Experimental Setup and Analytical Discussion

To verify the effectiveness of the proposed methodology about quantitative health assessment, the experimental test system of a multifunctional self-validating sensor was designed, which was sensitive to multiple physical parameters including temperature, humidity, and gas concentration. The experiment was conducted in a laboratory environment. The samples used to discuss in this section are actual sample data.

### 4.1. Multifunctional Sensor Experimental System

**Figure 7.**
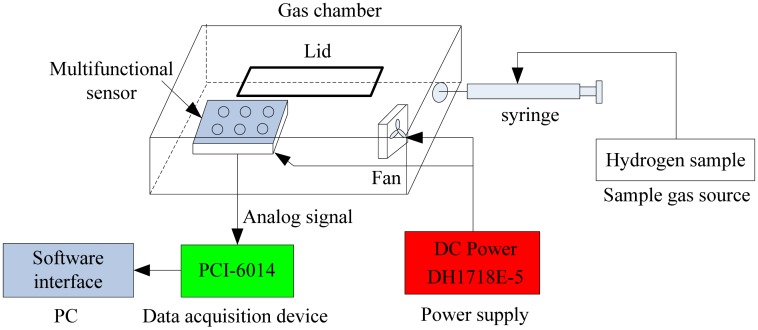
Block diagram of health evaluation experimental system.

The health evaluation software system was developed to read analog signals and store these source data in a file for further analysis. The system consisted of four key components: multifunctional sensor, data acquisition device, power supply and graphic user interface on PC. More details will be explained in the following sections. The block diagram of the health evaluation experimental system was shown in Figure 7.

The experiments were conducted in a sealed organic glass gas chamber (capacity is 50 cm × 20 cm × 10 cm). The multifunctional sensor was fixed in such a chamber. Hydrogen was selected as the gas sample and it was injected into the chamber by a syringe. There was a fan which assured the uniform distribution of the test gas in chamber.

#### 4.1.1. Multifunctional Sensor

The multifunctional sensor is composed of six sensitive units—four gas sensitive units, one temperature unit and one humidity unit, which are listed in Table 3, so it can be sensitive to the gas concentration, temperature, and humidity. To ensure valid measurements, the gas sensitive units with different sensitivity and working principles are selected.

**Figure 8.**
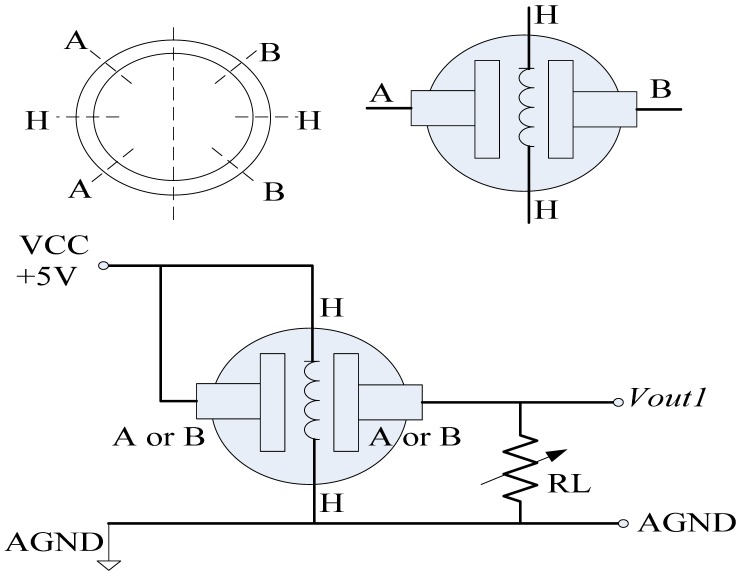
Structure and measuring circuit of semiconductor gas sensitive units.

In application of the semiconductor gas sensitive unit, both a heating circuit and a measuring circuit are required. A constant DC voltage is taken as heating signal to provide a certain elevated temperature; the semiconductor sensitive units are then sensitive to the detectable hydrogen. If hydrogen exits, its conductivity will increase. At this time, the conductivity changes will be converted into a measured voltage by using the corresponding measuring circuit. Its structure and measuring circuit are shown in Figure 8. Two terminal blocks H could be connected to the heating signal while A or B are the measured signal. The variable resistor RL is used to reflect the response under the detectable hydrogen, and the measured value could be obtained in voltage *Vout1*.

**Figure 9.**
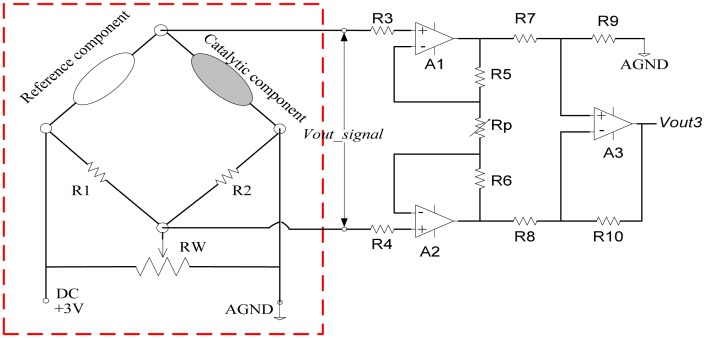
Measuring circuit of catalytic combustible gas sensitive units.

The catalytic combustible gas sensitive units consist of both catalytic and reference components. The corresponding measuring circuit is called a *Wheatstone bridge* as shown in the red box of Figure 9. In the presence of detectable hydrogen, it starts to burn on the surface of the catalytic component and a significant temperature rise is caused by the heat of combustion, which in turn leads into the resistance changes. The measuring bridge is then unbalanced, the offset *Vout_signal* is output as the measured signal, which rises linearly with the increase of detectable gas concentration. To avoid the negative influence of other factors during the combustion process, the reference component maintains a constant resistance. In operation, R1 and R2 are the precise resistors to keep the bridge balanced (usually R1 = R2). The variable resistor RW is used to adjust the zero position when there is no output.

To acquire the measured signal *Vout_signal*, a high common-mode rejection ratio circuit by using three amplifiers with high precision and low drift is designed as shown in Figure 9. The circuit could adjust the gain by the variable resistor Rp. To avoid the influence of offset current from amplifier, the resistances are configured as R3 = R4, R5 = R6, R7 = R8, R9 = R10. In this way, the preprocessed signal *Vout3* would vary linearly with *Vout_signal*, *i.e.*, the changes of detectable gas concentration. Denote the hydrogen concentration by *x*, and the undetermined coefficient by *a*, then:

(17) $$\begin{array}{c} \text{Vout}3=K\cdot x\\ K=a\cdot (R10/R8)\cdot (1+2\cdot R5/\text{Rp}) \end{array}$$

The LM35 and CHTM-02 are selected to detect the environmental parameters, respectively. Their outputs are linear with actual temperature and humidity. The former is 0–1V with temperature 0–100 centigrade while the latter is 1–3V with humidity 0–100%RH.

#### 4.1.2. Data Acquisition Device

For continuous acquisition of multiple outputs, the corresponding data acquisition software and hardware is necessary. These assignments are implemented on PC with a Windows XP operating system. A 16-channel PCI-data acquisition board with 16 bit accuracy (NI PCI-6014 Multifunction DAQ) was installed in PC and its sampling rate is up to 200 KHz. The acquisition program is written in Labview. A sampling rate of 2Hz for each channel was found to be the most appropriate for the experimental setup. The data acquisition software includes 6-channel data acquisition at real time, waveform display, data storage in a file and so on, which can provide enough samples to verify the *HRD* methodology.

#### 4.1.3. Measurement Protocol

The multifunctional sensor is first exposed to clean air for about 10 minutes to build a baseline environment for each sensitive unit. The zero position and amplification of the catalytic combustible measuring circuit are adjusted to ensure the data acquisition within its range. The hydrogen would be then injected into the chamber. The fan would help spread the gas sample to stability for about 5 minutes. The gas concentration could vary from 500 to 5,000 ppm. To simulate the broken heater strip of a semiconductor gas sensitive unit, the heating signal had been removed at once. The multifunctional sensor outputs would tend to be steady once more. After reaching a stable response, all the outputs would be sampled. The gas chamber needs to be cleaned again before the next experiment.

### 4.2. HRD Analysis of Multifunctional Self-validating Sensor

The *HRD* of multifunctional self-validating sensors means that the health evaluation is implemented from a global way and it is related to all the sensitive units. In this section, three situations which represent different health levels are introduced to interpret the proposed strategy.

#### Situation 1: all the sensitive units are fault free

**Figure 10.**
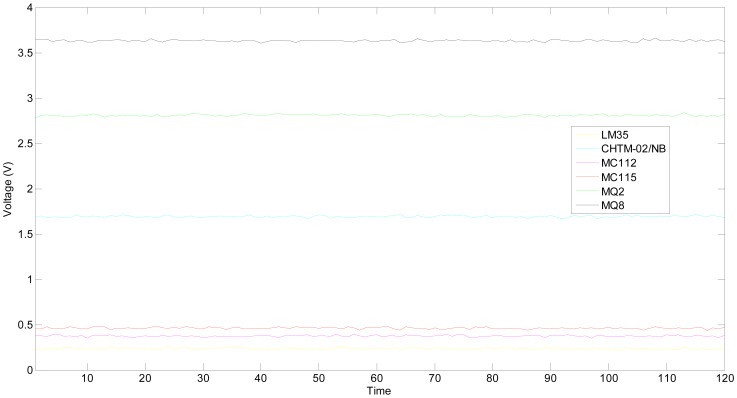
Static outputs of multifunctional self-validating sensor when it is fault free.

When the hydrogen concentration is 1,000 ppm, the static outputs of the multifunctional sensor are collected as shown in Figure 10. The temperature is about 23.7 °C and humidity is near 34.6%RH. Commonly, the health state should be in *HS* or *SH*, and the validity of the proposed *HRD* methodology is then evaluated. By using fault free samples in the calibration, the best estimation *μ_HS_* of the true values of all sensitive units as well as their base-level values Δ are shown in Table 4.

The grey whitening function parameters are defined in Table 5, which represent the extent of deviation from the health values or the best estimation *μ_HS_*.

To illustrate the proposed *HRD* methodology, the first sample of Figure 10 is taken as an example and their outputs are given in Table 6.

By using Equation (7), the grey sample evaluating matrix *GSE*_1_ at the first measurement point is constructed as:

(18) $${\text{GSE}}_{1}=\left[\begin{array}{cccc} 0.9577 & 1.0000 & 0.2759 & 0.0000\\ 1.0000 & 0.5033 & 0.0336 & 0.0000\\ 1.0000 & 0.5241 & 0.2184 & 0.0000\\ 0.9964 & 0.8682 & 0.3617 & 0.0000\\ 0.9487 & 0.9944 & 0.8874 & 0.0000\\ 0.9824 & 1.0000 & 0.5373 & 0.0000 \end{array}\right]$$

By using Equations (12–15), the weight vector of six sensitive units is also computed based on the information entropy method:

(19) $$W_{1}=(w_{11},w_{21},\ldots ,w_{61})=[0.1631 0.2647 0.1795 0.1465 0.1162 0.1299]$$

By using Equations (16), the comprehensive grey assessment values (*CGAV*_1_) can be computed:

(20) $${\text{CGAV}}_{1}=[\;\begin{array}{cccc} 0.9843 & 0.7631 & 0.3190 & 0.0000 \end{array}\;]$$

From the traditional qualitative health evaluation idea, the maximum of the *CGAV*_1_s is picked out and then the current health state is determined to be in *HS*. By using the *HRD* computing formula as shown in Equation (1), the quantitative health level is achieved through the data fusion of the *CGAV*_1_:

(21) $${\text{HRD}}_{1}=f({\text{CGAV}}_{1})=0.9251$$

**Figure 11.**
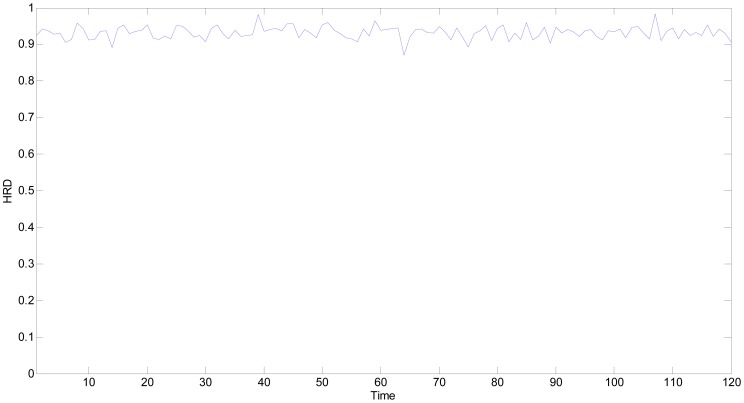
*HRD*s of multifunctional self-validating sensor when it works normally.

In a similar way, the *HRD*s of all the time points are computed as shown in Figure 11. The fluctuation of *HRD* curve is mainly caused by the measurement noise. In Figure 11, all the *HRD*s are greater than 0.90, excluding the 64th time point (its *HRD* equals to 0.8703), which implies that the multifunctional self-validating sensor is either *HS* or *SHS*. The experimental results are consistent with the normal operational condition, which validates the proposed method.

The *HRD* of the 64th time point is lower than 0.9, therefore, further research is needed and the measurement outputs are shown in Table 7. The output of temperature sensitive unit LM35 has deviated 1.9 °C from the 23.7 °C setpoint, which has exactly caused the health level degradation of the whole multifunctional self-validating sensor.

#### Situation 2: one sensitive unit is faulty

**Figure 12.**
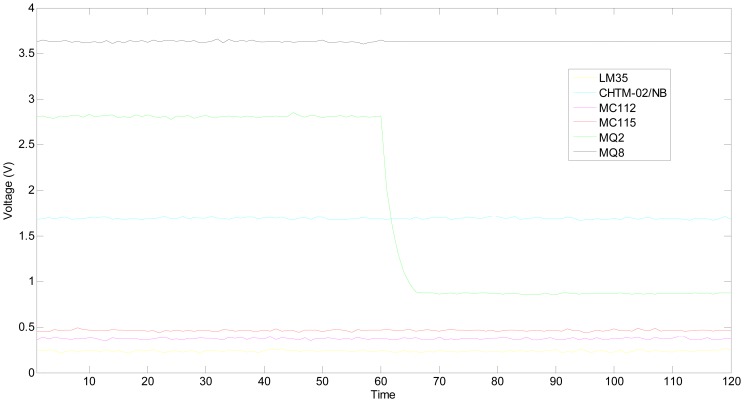
Static outputs when MQ2 is faulty.

This experiment was done in the same gas chamber on the same day, and the heating voltage of MQ2 was removed to simulate that a broken heater strip at the 61th time point. After about 7 seconds, its output tends towards becoming steady again. The hydrogen concentration is still 1,000 ppm, and the outputs of multifunctional self-validating sensor are shown in Figure 12.

**Figure 13.**
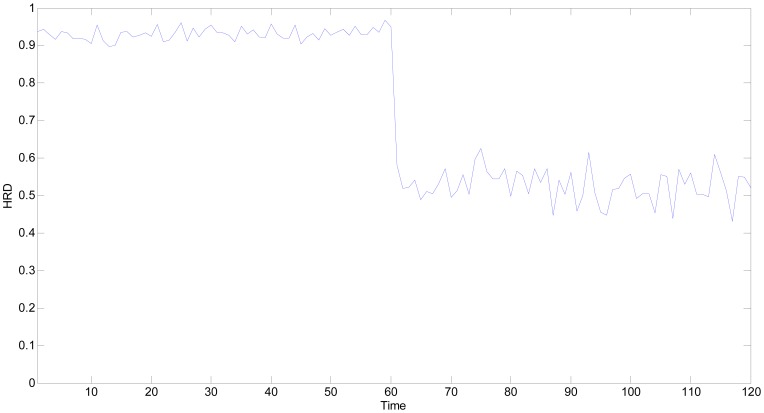
*HRD*s of multifunctional self-validating sensor when MQ2 is faulty.

The experimental environment is the same as situation 1, therefore, the best estimation of true values of all sensitive units and grey whitening function parameters are also the same as those given in Tables 4 and 5. By using the proposed *HRD* strategy, the *HRD*s of all the time points under single fault can be obtained as shown in Figure 13.

In Figure 13, all the *HRD*s are lower than 0.70 starting from the 61th time point, which implies that the multifunctional self-validating sensor is in *MF*. The experimental results are consistent with the proposed extended meaning of *HRD*.

#### Situation 3: more sensitive units are faulty

**Figure 14.**
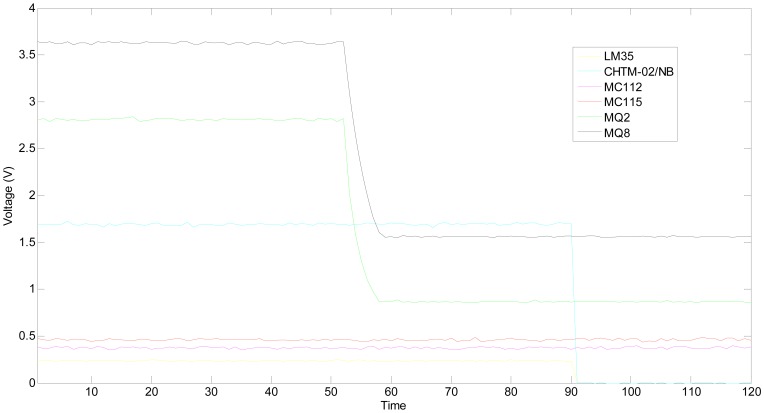
Static outputs when more sensitive units are faulty.

This experiment has also been done in the same gas chamber on the same day, and the heating voltage of MQ2 and MQ8 would be both removed to simulate that both heater strips are broken. The moment when faults occur is at about the 51th time point. After about 8.5 seconds, their outputs tend towards a steady value again. The hydrogen concentration is still 1,000 ppm, and the static outputs of the multifunctional self-validating sensor are shown in Figure 14. To simulate more faults, the power supply of temperature and humidity sensitive unit was also removed at the 91th time point and most sensitive units are then faulty.

**Figure 15.**
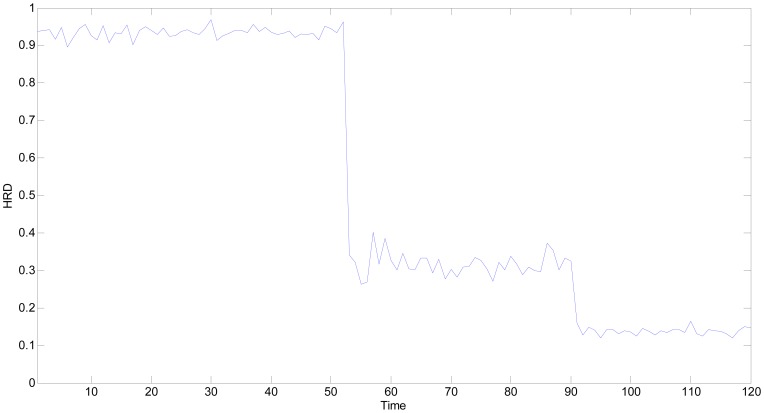
*HRD*s of multifunctional self-validating sensor when more sensor units are faulty.

By using the proposed *HRD* strategy, the *HRD*s of all the time points under multiple faults are shown in Figure 15. In Figure 15, the *HRD*s have decreased to nearly 0.3 starting at the 51th time point, which is caused by the two faulty sensitive units. According to the definition of *HRD*, the current health state is still in *MF*, which agrees with the experimental situation. The measured results of MQ2 and MQ8 are both unbelievable, and the overall health level has become very low and it has degraded further than the above situation 2.

With more faulty sensitive units, the *HRD* is bound to be lower and the measured output would also be more suspect. In the latter part of Figure 14 starting from about the 91th point, the four sensitive units have been deemed faulty. The corresponding *HRDs* have further decreased to nearby 0.1 as shown in Figure 15. The current health state is in *FS*, the multifunctional self-validating sensor should be exchanged because most of sensitive units have suffered failures.

### 4.3. HRD Analysis of Single Sensitive Unit

In this section, two situations which imply different health levels are introduced to interpret the health evaluation of a single sensitive unit from the local way.

#### Situation 1: single sensitive unit is fault free

The actual experimental sample of MQ2 is still the one from Figure 10, and the best estimation of its true value and grey whitening function parameters are the same as Tables 4 and 5. In the process of computing *HRD* of single sensitive unit, the length of time point series will be selected as six points to simplify the calculation, because it is equal to the number of sensitive units.

The sample is updated online by the latest measurement. In other words, the latest output would be added to the sample, and the sample at the first time point is removed. Taking the first six measurements in Figure 10 as an example to illustrate the *HRD* of a single sensitive unit in detail, their outputs are shown in Table 8. The latest or current measurement is 2.7937V.

Firstly, by using the Equations (8–11), the scaling value *d_ij_*, comparison matrices *CM_i_*, largest eigenvalue *λ*_max_, the corresponding eigenvector *α*_max_ and the *CR* are all obtained:

(22) $$d_{\text{MQ}2}=[0.0262 0.0020 0.0011 0.0042 0.0017 0.0161]$$

(23) $${\text{CM}}_{\text{MQ}2}=\left[\begin{array}{llllll} 1.0000 & 13.3341 & 22.9753 & 6.2731 & 15.1051 & 1.6207\\ 0.0750 & 1.0000 & 1.7230 & 0.4705 & 1.1328 & 0.1215\\ 0.0435 & 0.5804 & 1.0000 & 0.2730 & 0.6575 & 0.0705\\ 0.1594 & 2.1256 & 3.6625 & 1.0000 & 2.4079 & 0.2583\\ 0.0662 & 0.8828 & 1.5210 & 0.4153 & 1.0000 & 0.1073\\ 0.6170 & 8.2276 & 14.1766 & 3.8707 & 9.3204 & 1.0000 \end{array}\right]$$

(24) $$\lambda_{\max}=6.000,\alpha_{\max}=[-0.8388-0.0630-0.0366-0.1339-0.0556-0.5182];$$

(25) CR=0<0.10

The above *α*_max_ can serve as the weight distribution because *CR* is less than 0.10. By normalizing the eigenvector *α*_max_, the weight *W_MQ2_* is then obtained based on the *AHP* method:

(26) $$W_{\text{MQ}2}=(w_{51,}w_{52},\ldots ,w_{56})=[0.5099 0.0382 0.0222 0.0813 0.0338 0.3146]$$

Secondly, by using Equations (6), the grey sample evaluating analysis matrix *GSE_MQ2_* is computed:

(27) $${\text{GSE}}_{\text{MQ}2}=\left[\begin{array}{cccc} 0.9487 & 0.9944 & 0.8874 & 0.0000\\ 1.0000 & 0.1703 & 0.0697 & 0.0000\\ 1.0000 & 0.0858 & 0.0351 & 0.0000\\ 1.0000 & 0.3527 & 0.1443 & 0.0000\\ 1.0000 & 0.1513 & 0.0619 & 0.0000\\ 0.9798 & 1.0000 & 0.5488 & 0.0000 \end{array}\right]$$

Then, by using Equation (16), the comprehensive grey assessment values (*CGAV*) are then obtained:

(28) $${\text{CGAV}}_{\text{MQ}2}=[\;\begin{array}{cccc} 0.9675 & 0.8638 & 0.6424 & 0.0000 \end{array}\;]$$

Lastly, by using Equation (1), the *HRD* of MQ2 is calculated:

(29) $${\text{HRD}}_{\text{MQ}2}=f({\text{CGAV}}_{\text{MQ}2})=0.9067$$

**Figure 16.**
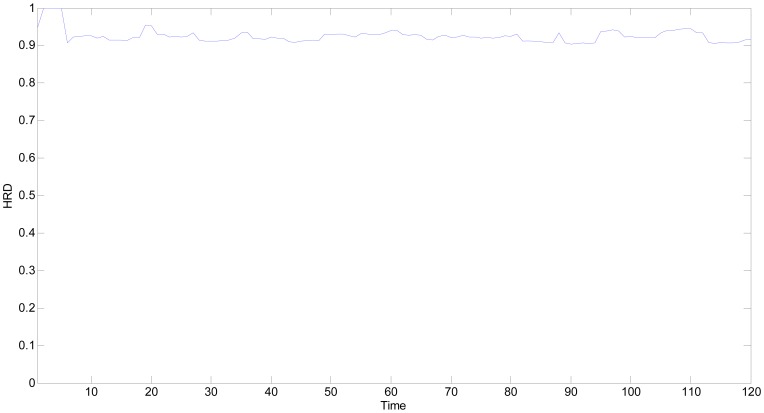
*HRD*s of fault free MQ2.

In a similar way, the *HRD*s of MQ2 under a fault free state are calculated as shown in Figure 16. The first five time points could not construct the multi-point time series, their *HRD*s would be replaced by the values of *HS* whitening function. In Figure 16, all the *HRD*s are greater than 0.9, which verifies the proposed *HRD* methodology under normal operational conditions.

#### Situation 2: single sensitive unit is faulty

**Figure 17.**
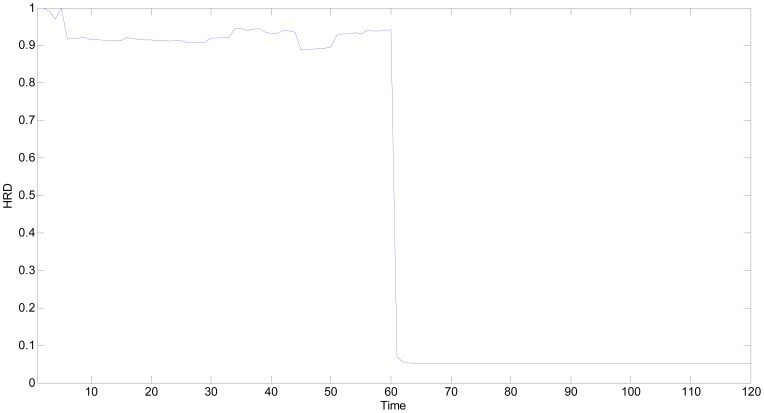
*HRD*s of faulty MQ2.

The actual experimental samples of faulty sensitive unit MQ2 are from Figure 12. The heating voltage of MQ2 at the 61th time point is removed and the *HRD* results are shown in Figure 17.

The sensitivity of the measured outputs is the key in the proposed *AHP* based weight distribution, and the larger weight will be distributed to the point whose output is close to *FS*. The guideline rightly conforms to the health evaluation of a ingle sensitive unit, because *HRD* should have a fast response to faults. In Figure 17, the *HRD* has started to decline rapidly starting from the 61th time point, which is consistent with the removal of the heating voltage. Once faults occur, the *HRD* is lower than 0.1 and the MQ2 is in *FS*. The proposed *HRD* methodology is verified again under faults and it could thus also be treated as a tool of fault detection.

## 5. Conclusions

This paper has presented the design of a health evaluation system for multifunctional self-validating sensors by using two semiconductor gas sensitive units, two catalytic combustible gas sensitive units, one temperature and one humidity sensitive units. The novel concept of *HRD* is proposed to describe the health level in a quantitative way, and its inner and extended meanings are explained in detail. The emphasis of this article lies on the *HRD* methodology itself by using multi-variable data fusion technology coupled with a grey evaluation algorithm. To get the valid weights distribution of all sensitive units and the sensitivity of different time points, the information entropy and *AHP* method are used, respectively. The *HRD* takes the correlation of multiple parameters into consideration.

The experimental system of the multifunctional self-validating sensor was designed in the laboratory. From the “local” and “global” viewpoints, the *HRD* of an overall multifunctional sensor at a single time point and a single sensitive unit at multiple time points were analyzed thoroughly. Based on actual samples, three situations of the multifunctional sensor and two situations of a single sensitive unit have been considered in the *HRD* application, which represent different health levels. The results show that the proposed *HRD* can be used to indicate the quantitative health level and it rapidly reflects the performance changes of a multifunctional sensor.

As one of the most important self-validating functions, the health evaluation is rather meaningful. The following work will include two aspects: one is the health forecasting based on historical *HRD* information; the other is the fault recovery to improve the *HRD*.

## Figures and Tables

**Table 1.** Relationship between *HRD* and health degradation stages.

	***HRD***	**Health stages**
1	[0.9, 1.0]	*HS*
2	[0.7,0.9)	*SH*
3	[0.2,0.7)	*MF*
4	[0.0, 0.2)	*FS*

**Table 2.** *RI* values.

***n***	**1**	**2**	**3**	**4**	**5**	**6**	**7**	**8**	**9**	**10**	**11**	**12**	**13**	**14**	**15**
*RI*	0	0	0.52	0.89	1.12	1.26	1.36	1.41	1.46	1.49	1.52	1.54	1.56	1.58	1.59

**Table 3.** Lists of sensitive units.

**Sensitive units**	**Description**
MQ-2	Semiconductor
MQ-8	Semiconductor
MC112	Catalytic combustible
MC115	Catalytic combustible
LM35	Temperature
CHTM-02	Humidity

**Table 4.** Best estimation of true values and base-level values.

	**LM35**	**CHTM-02**	**MC112**	**MC115**	**MQ2**	**MQ8**
*μ_HS_* (V)	0.2370	1.6930	0.3750	0.4620	2.8099	3.6299
Δ	0.01 °C	0.01%RH	1 ppm	1 ppm	1 ppm	1 ppm

**Table 5.** Whitening function parameters of the grey model.

	***HS* (M_1_)**	***SH* (M_2_)**	***MF* (M_3_)**	***FS* (M_4_)**
LM35	[8.0,8.0]	$\left[\begin{array}{cc} 100 & 10\\ 10 & 100 \end{array}\right]$	$\left[\begin{array}{cc} 500 & 150\\ 150 & 500 \end{array}\right]$	[800,800]
CHTM-02	[5.5,5.5]	$\left[\begin{array}{cc} 100 & 10\\ 10 & 100 \end{array}\right]$	$\left[\begin{array}{cc} 500 & 150\\ 150 & 500 \end{array}\right]$	[800,800]
MC112	[20,20]	$\left[\begin{array}{cc} 50 & 25\\ 25 & 50 \end{array}\right]$	$\left[\begin{array}{cc} 200 & 60\\ 60 & 200 \end{array}\right]$	[500,500]
MC115	[20,20]	$\left[\begin{array}{cc} 50 & 25\\ 25 & 50 \end{array}\right]$	$\left[\begin{array}{cc} 200 & 60\\ 60 & 200 \end{array}\right]$	[500,500]
MQ2	[20,20]	$\left[\begin{array}{cc} 50 & 25\\ 25 & 50 \end{array}\right]$	$\left[\begin{array}{cc} 200 & 60\\ 60 & 200 \end{array}\right]$	[500,500]
MQ8	[20,20]	$\left[\begin{array}{cc} 50 & 25\\ 25 & 50 \end{array}\right]$	$\left[\begin{array}{cc} 200 & 60\\ 60 & 200 \end{array}\right]$	[500,500]

**Table 6.** First static measurements in Figure 10.

**Sensitive units**	**LM35**	**CHTM-02**	**MC112**	**MC115**	**MQ2**	**MQ8**
Output (V)	0.2328	1.6940	0.3799	0.4518	2.7837	3.6430

**Table 7.** Static outputs of 64th time point.

**Sensitive units**	**LM35**	**CHTM-02**	**MC112**	**MC115**	**MQ2**	**MQ8**
Output (V)	0.2181	1.7158	0.3634	0.4386	2.8164	3.6116

**Table 8.** Measured outputs of MQ2 at first six points.

**Time point series**	**1**	**2**	**3**	**4**	**5**	**6**
Output (V)	2.7837	2.8079	2.8110	2.8057	2.8081	2.7937
